# Vascular access in Pará: clinical and epidemiological aspects of patients on renal replacement therapy at the largest specialist center in Pará state, Brazil

**DOI:** 10.1590/1677-5449.202400522

**Published:** 2026-04-27

**Authors:** José Maciel Caldas dos Reis, Iara de Brito Silva, Maria Gabriela Perdigão Barros Monteiro, Maria Mirella Nunes Bessa Guerra, Daniele Lima da Costa, Fabiany de Fátima Pompeu Rodrigues, Mariseth Carvalho de Andrade

**Affiliations:** 1 Hospital de Clínicas Gaspar Vianna – HCGV, Belém, PA, Brasil.; 2 Centro Universitário Metropolitano da Amazônia – UNIFAMAZ, Belém, PA, Brasil.; 1 Hospital de Clínicas Gaspar Vianna – HCGV, Belém, PA, Brasil.; 2 Centro Universitário Metropolitano da Amazônia – UNIFAMAZ, Belém, PA, Brasil.

**Keywords:** kidney failure, renal dialysis, epidemiology, renal replacement therapy

## Abstract

**Background:**

There are no official data on the prevalence of end-stage chronic kidney disease with dialysis for the Brazilian state of Pará.

**Objectives:**

To describe the clinical-epidemiological profile of hemodialysis patients at the largest specialist center in the state of Pará.

**Methods:**

This is an observational, cross-sectional, descriptive, quantitative study. Simple descriptive statistics were calculated. Data were collected from medical records from June to August 2022 at the Hemodialysis Center at the Hospital de Clínicas Gaspar Vianna (HCGV) and the Monteiro Leite Hemodialysis Clinic (CMHL), both in Belém, Pará, Brazil. A total of 191 patients from the chronic hemodialysis program were interviewed.

**Results:**

Of the total sample, 28.8% patients were from HCGV and 71.6% from CHML; 57.1% were men and 42.9% were women. Mean age was 54.1 years. Results showed that 65.4% of patients self-reported skin color as brown, 44.5% had completed primary education, 41.9% were single, 71.4% had hypertension, 40.6% had diabetes, and 77.5% had been on renal replacement therapy for a mean time of 4 years. Regarding treatment, 86.9% started treatment with a short-duration catheter, 8.4% were using a long-dwelling catheter, and 4.7% had a definitive dialysis access via a mature arteriovenous fistula (AVF). Currently, 58.1% of these patients have a native AVF, 34% have a long-dwelling catheter, and 3.1% have a short-duration catheter and no AVF.

**Conclusions:**

Patients on renal replacement therapy in Pará predominantly initiate hemodialysis in an unplanned manner, using temporary venous catheters, although they transition to definitive vascular access over time. This scenario highlights the need for early diagnosis of chronic kidney disease and timely vascular access planning, aiming to reduce complications and optimize clinical outcomes.

## INTRODUCTION

Chronic kidney disease (CKD) is a global public health problem, and its main causes are arterial hypertension and diabetes mellitus (DM) in the most diverse of populations.^1^ In 2012, the International Society of Nephrology updated the clinical definition of CKD to a glomerular filtration rate (GFR) below 60 mL/min/1.73 m^2^ for at least 3 months, irrespective of cause. In turn, end-stage kidney disease (ESKD) is defined as when estimated GFR is less than 15 mL/min/1.73 m^2^, indicating kidney failure requiring dialysis.^2^

End-stage chronic renal failure (CRF) is characterized by deterioration of the biochemical and physiological functions of all of the body’s systems, occurring when the kidneys are incapable of removing the products of metabolic degradation from the body or perform their regulatory functions.^1-3^

In conjunction with appropriate treatment of kidney diseases, screening can delay disease development and progression to loss of renal properties.^1,2^ The population aging and increased life expectancy that have resulted from demographic transition over recent decades in Brazil have contributed to changes in the profile of morbidity and mortality and increased the prevalence of chronic diseases, including CKD.^3,4^

Central venous catheters (CVC) have become permanent components of the hemodialysis process, particularly for patients who undergo dialysis cycles three times a week. However, incorrect practices and handling of CVCs contribute to infectious processes, both site-specific and systemic, which can cause serious complications, such as septicemia.^4,5^

In Brazil, the most recent data, from the 2020 Brazilian Dialysis Census, confirm the tendency observed over recent years in relation to the increase in the number of patients on dialysis, with an estimated total in the order of 144,779. It was estimated that there were 44,264 new dialysis patients in 2020, and the estimated prevalence of dialysis patients in Brazil’s North administrative region is 283 per million population (pmp), with a total incidence of 209 pmp, which exceeds rates for Latin America (159 pmp) and Europe (122 pmp) from 2018, but is lower than the 2017 figure for the United States (370 pmp).^1,5,6^

There are no official data for the prevalence of ESKD requiring chronic dialysis in the Brazilian state of Pará. A survey conducted for the state’s CKD patient care plan, published in May of 2015, listed 2,692 patients on renal replacement therapy (RRT) in Pará, at 23 RRT services registered on the country’s National Healthcare Establishments Register (CNES - Cadastro Nacional de Estabelecimentos de Saúde) that year. As such, care was provided to around half of the population of patients needing RRT, despite the increases in provision of RRT services and hemodialysis capacity in Pará state that have occurred since 2011.^4^

The growing number of patients with CKD represents a significant challenge for the health care system, since, in addition to the progressive increase in public health expenditure, it is associated with high rates of morbidity and mortality.^7-9^

Thus, the objective of the present study is to describe the clinical and epidemiological profile of patients on RRT at the largest specialist hemodialysis center in the Brazilian state of Pará.

## METHODS

### Study design

This is a cross-sectional, retrospective, observational, descriptive study, using a quantitative approach and based on documentary fieldwork. The methodology employed adheres to the principles set out in the Guidelines on Good Publication Practice, published by the Committee on Publication Ethics (COPE)^10^ . Data were collected from June to August 2022, during hemodialysis sessions at the Hemodialysis Center at the Hospital de Clínicas Gaspar Vianna (HCGV) and the Hemodialysis Clinic at the Hospital Monteiro Leite (CHML), both located in Belém, Pará, Brazil. Specific clinical data were extracted from patient medical records available on site. We interviewed 191 out of a total of 214 patients on the institutions’ chronic hemodialysis program.

The patients answered a standardized semi-structured questionnaire with the help of a researcher who collected data at the hemodialysis centers. All patients, or their legal guardians, signed free and informed consent forms granting permission for scientific publication of their data.

### Ethical considerations

The project was submitted in advance to the Research Ethics Committee with Ethics Appraisal Submission Certificate (CAAE) number 00497817.4.0000.5701 and complies with National Health Council Resolution (Conselho Nacional de Saúde) 466/2012 ensuring confidentiality and anonymity. The project was approved by the ethics committee with substantiated opinion number 2.954.5541.

### Study population

**Figure 1:**
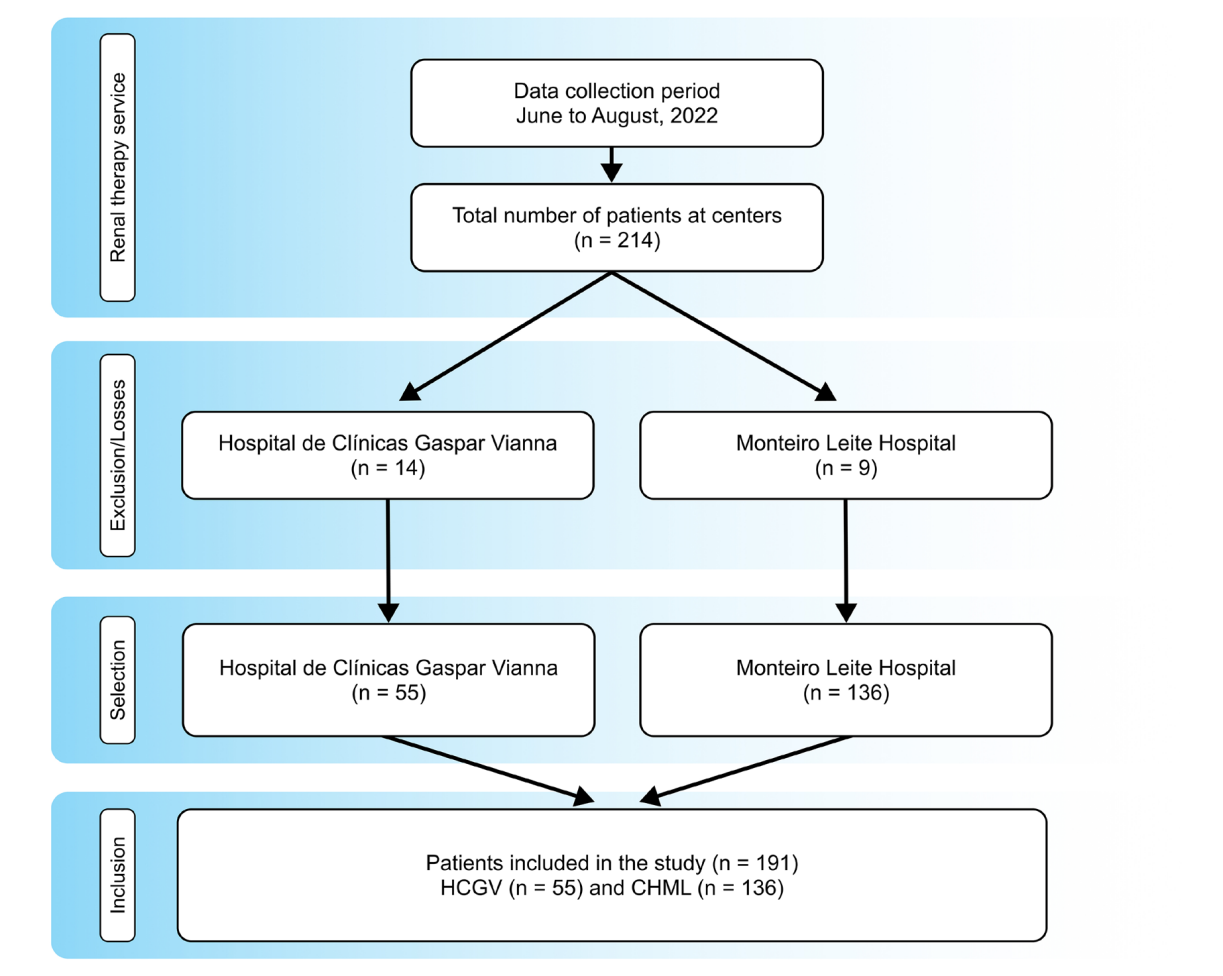
Flow diagram illustrating selection, losses, and inclusion of patients in the study.

The study enrolled all chronic kidney dialysis patients registered at the institutions who agreed to take part, signed the free and informed consent forms, were over the age of 18 years, of both sexes, and were on the HCGV and CHML’s chronic hemodialysis programs. Patients were excluded who were on peritoneal dialysis or for whatever reason did not attend their hemodialysis sessions on data collection days, as detailed in the STROBE flow diagram shown in Figure 1.

### Sample size calculation

With the objective of describing the clinical and epidemiological profile of RRT patients at the largest specialist center in the state of Pará, a sample size calculation was conducted for a 95% confidence level (Z = 1.96), 5% acceptable error, and expected proportion of 50%. Based on the population served, 214 RRT patients, the final sample size was 130 individuals, according to the formula described below. The calculation used the methodology proposed by Luchesa and Chaves.^11^

### Data collected

Data were collected using an instrument containing structured questions covering patients’ sociodemographic data (age, sex, marital status, ethnicity, and educational level) and clinical factors (variables related to underlying diseases related to hemodialysis and types of access). The investigation of intercurrent hemodynamic, infectious, and metabolic conditions related to vascular access or other systemic complications during hemodialysis was restricted to events occurring during the 6 months prior to data collection.

### Statistical analysis

Data were analyzed using descriptive statistics, expressed as absolute and relative frequencies (percentages) for categorical variables. Comparisons between proportions were performed using Pearson’s chi-square test. A significance level of α = 0.05 (5%) was adopted, and p-values < 0.05 were considered statistically significant and indicated with an asterisk (*). Data organization and analysis were performed using Microsoft Excel®.

## RESULTS

Analysis of the patients’ socioeconomic profile enabled aspects of their clinical and social situation to be identified. A total of 191 patients were interviewed, 55 (28.8%) from the HCGV and 136 (71.6%) from the CHML. With regard to sex distribution, 57.1% patients were male and 42.9% were female. With regard to age, the average age was 54.1 years, ranging from 18 to 93 years. The two highest prevalence rates were for the age brackets 50 to 59 and 60 to 69 years. The predominant self-report skin color was brown (65.4%) (Table 1).

**Table 1:** Patients’ demographic characteristics.

**Variables**	**N**	**%**
Sex		
Female	82	42.9
Male	109	57.1
Age group^*^ (years)		
< 30	20	10.5
30 to 39	14	7.3
40 to 49	33	17.3
50 to 59*	47	24.6
60 to 69*	42	22.0
≥ 70	35	18.3
Skin color *		
Brown*	125	65.4
Black	35	18.3
White	31	16.2

* p = 0.0001; Chi-square test.

With regard to educational level, 44.5% of the patients stated they had completed primary education. The marital status of 41.9% of the sample was single. Nutritional status was considered adequate in 46.1%, with just 8.4% classed as underweight. Data on the patients’ economic profile revealed that 83.3% had a monthly income in the range of 1 to 3 times the minimum wage and 48.4% of interviewees were receiving government disability benefits. The great majority of the patients (94.3%) lived with their families, 80.2% were living in their own homes, and 58.9% were from Belem, the Pará state capital. A substantial proportion of patients resided in inland municipalities of the state and in the metropolitan region, totaling 41.1%. (Tables 2 and 3).

**Table 2:** Variables related to patients’ educational level, marital status, and nutritional classification.

**Variables**	**N**	**%**
Educational level^*^		
No education	11	5.8
Primary education not completed	19	9.9
Completed primary education*	85	44.5
Secondary education	57	29.8
Higher education	19	9.9
Marital status*		
Single*	80	41.9
Married*	76	39.8
Divorced	19	9.9
Widowed	16	8.4
Nutritional status *		
Underweight	16	8.4
Healthy weight *	88	46.1
Overweight	56	29.3
Obese	22	11.5
Data missing	9	4.7

* p = 0.0001; chi-square test.

**Table 3:** Patients’ socioeconomic data.

**Variables**	**N**	**%**
Income (MWs)^*^		
No income	4	2.1
Less than 1	6	3.1
1 to 3*	160	83.3
Greater than 3	6	3.1
Did not know	15	7.8
Source of income*		
Receives disability benefit*	93	48.4
Retired	81	42.2
Waiting for benefit/pension	33	17.2
Still working	22	11.5
Residence status*		
Living with family*	181	94.3
Living alone	11	5.7
Housing status*		
Own home*	154	80.2
Rental	27	14.1
Ceded	11	5.7
Origin*		
State capital*	113	58.9
Metro area	21	10.9
State interior	58	30.2
Transport for hemodialysis*		
Provided by city	69	36.1
Other type of transport *	122	63.9

* p = 0.0001; chi-square test.

MWs = multiples of minimum wage.

Causes of CKD included systemic arterial hypertension (SAH), responsible for 39.1% of the patients developing the disease, and type 2 DM, which caused CKD in 34.4%. The most prevalent comorbid diseases were also SAH and DM, present in 71.4% and 40.6%, respectively. With regard to risk factors, the most important were alcoholism, smoking, and obesity. Eighty-five (44.3%) of the interviewees drank alcoholic beverages and 67 (34.9%) had smoked tobacco (Table 4).

**Table 4:** Patients’ risk factors associated with chronic kidney disease.

**Variables**	**N**	**%**
Causes of chronic kidney disease^*^		
Hypertension*	75	39.1
Type 2 diabetes mellitus*	66	34.4
Indeterminate cause	32	16.7
Systemic lupus erythematosus	10	5.2
Polycystic disease	8	4.2
Kidney stones	8	4.2
Glomerulonephritis	4	2.1
Other causes	49	25.5
Comorbidities*		
Systemic arterial hypertension*	137	71.4
Diabetes mellitus	78	40.6
Rheumatic diseases	45	23.4
Stroke	41	21.4
Kidney stones	38	19.8
Dyslipidemia	35	18.2
Acute myocardial infarction	30	15.6
Glomerulonephritis	18	9.4
Obstructive uropathies	12	6.3
Cancer	2	1.0
Risk factors		
Alcoholism	85	44.3
Smoking	67	34.9
Obesity	26	13.5

* p = 0.0001; Chi-square test.

It is important to point out that 169 (88.5%) of the patients had not had a biopsy and just 49 (25.7%) patients had undergone conservative treatment, a minority of whom (26.5%) had received conservative treatment for less than 6 months, 30.6% had undergone it for 6 to 12 months, and a majority (40.9%) had received conservative treatment for more than 1 year.

It was found that most patients initiated RRT using short-term double-lumen catheters (DLC). It was also observed that the majority of patients later transitioned to an arteriovenous fistula (AVF) or a long-dwelling CVC, depending on their indications for RRT and their clinical condition. An AVF is the preferred access for patients who will require RRT indefinitely. Differences in AVF site and number of procedures performed were not analyzed (Tables 5 and 6).

**Table 5:** Venous access methods at start and end of study in patients on renal replacement therapy in Pará, Brazil.

**Variables**	**N**	**%**
Short duration DLC^*^	166	86.9
Long-dwelling permacath	16	8.4
AVF	9	4.7
Current vascular access*		
Native AVF*	111	58.1
Long-dwelling catheter	65	34.0
AVF with PTFE	9	4.7
Short-duration DLC	6	3.1

* p = 0.0001; chi-square test.

DLC = double-lumen catheter; AVF = arteriovenous fistula; PTFE = polytetrafluoroethylene.

**Table 6:** Site and method of access in patients on renal replacement therapy in Pará, Brazil.

**Variables**	**N**	**%**
DLC site		
Right SCV	3	50.0
Right IJV	2	33.3
Right CFV	2	33.3
Number of DLCs fitted		
One	1	16.7
Two	4	66.7
Three or more	1	16.7
Long-dwelling catheter site^*^ (n = 65)		
Right IJV *	23	35.4
Right SCV	17	26.2
Left SCV	11	16.9
Left IJV	8	12.3
Left CFV	5	7.7
Right CFV	1	1.5
Number of long-term dialysis catheters		
One*	36	55.4
Two	15	23.1
Three	6	9.2
Four or more	8	12.3
AVF type* (n = 120)		
Left BC*	44	36.7
Right BC*	34	28.3
Left BB	18	15.0
Left RC	17	14.2
Right BB	10	8.3
Right RC	6	5.0
Number of AVFs created*		
One*	82	67.2
Two	25	20.5
Three	6	4.9
Four or more	6	4.9

* p = 0.0001; chi-square test.

BB = brachiobasilic; BC = brachiocephalic; DLC = double-lumen catheter; AVF = arteriovenous fistula; RC = radiocephalic; CFV = common femoral vein; IJV = internal jugular vein; SCV= subclavian vein.

The majority of patients in the sample were interested in receiving a kidney transplant, although transplantation was not indicated in all cases (Table 7). With regard to hospital admissions, approximately 68% of the patients needed to be admitted at some point, with hypertensive emergencies being the most common cause reported (Table 8).

**Table 7:** Factors related to kidney transplantation and understanding of the disease.

**Variables**	**N**	**%**
Interested in kidney transplantation^*^	125	65.4
In preparation for transplantation	69	36.1
Prior knowledge of the disease	42	22.0
Family history of dialysis	37	19.4

* p = 0.0001; chi-square test.

**Table 8:** Admissions and their causes among patients on renal replacement therapy in Pará, Brazil.

**Variables**	**N**	**%**
Hospital admissions^*^		
Yes*	129	67.5
No	42	22.0
Cause of admission* (n = 129)		
Hypertensive emergencies*	108	77.1
Other infections	86	44.8
Metabolic disorders	77	40.1
Access-related infection	64	33.3
Cardiovascular events	50	26.0
Hypervolemia	25	13.0

* p = 0.0001; chi-square test.

## DISCUSSION

The present study analyzed the questionnaire developed by the researchers, completed by 191 volunteer patients enrolled in the chronic hemodialysis program at HCGV and CHML. Data analysis demonstrated a higher prevalence of male patients, a finding consistent with the literature, which associates this predominance with greater exposure to modifiable risk factors, such as alcohol consumption, smoking, obesity, and physical inactivity, as well as lower adherence to preventive and health-promoting measures compared with female patients.^9,12^

Regarding the mean age observed among the patients, this finding is consistent with the age profile described in the 2020 Brazilian Society of Nephrology Census, which demonstrates a higher concentration of individuals in older age groups among patients undergoing RRT.^1^ With respect to skin color, the results should be interpreted in light of regional demographic characteristics, particularly considering the predominant population composition in the state of Pará and the Northern region of Brazil.^4^

The most prevalent educational level observed demonstrates that access to education in terms of knowledge about disease makes it more likely that patients will seek treatment early. It is also understood that patients with CKD very often need treatment for extended periods, which is tiring for both patients and their support networks, which may explain the high proportion of single patients receiving treatment.^12^

Maintaining a healthy weight during treatment, which was observed in a majority of patients, is a positive factor that reduces complications and hospital admissions, particularly since it is known that RRT has harmful effects on metabolism, particularly with regard to proteins, and can cause malnutrition, with worse clinical outcomes.^13^

The reported income is in line with the average monthly income observed in the Brazilian population in 2022, according to data from the Continuous National Households Sample Survey (PNAD Contínua - Pesquisa Nacional por Amostra de Domicílios) of the Brazilian Institute of Geography and Statistics (IBGE - Instituto Brasileiro de Geografia e Estatísticas),^14^ particularly related to government welfare payments, which supplement income and help to cover basic costs. Living with one’s family provides emotional and logistic support to sustain the long-term dialysis treatment.

Data from the Brazilian Society of Nephrology Census indicate that SAH and DM remain the leading causes of CKD in Brazil.^1^ This etiological profile is consistent with national and international literature, which recognizes these conditions as the main determinants of CKD progression and the need for RRT.^3,9^ Furthermore, the high prevalence of cardiovascular and metabolic diseases in the population contributes to the persistence of this epidemiological pattern.

Among the risk factors observed, consumption of alcoholic beverages was reported by around half of the interviewees, which, when consumed in high doses, can cause elevated energy intake and, in common with excess weight, increases arterial blood pressure and cardiovascular risk, provoking progression of CKD. Similarly, tobacco use is associated with increased albuminuria, which progressively worsens renal damage.^6^

The phenomenon of initial treatment using a short-term DLC is notable because of the oligosymptomatic progression of kidney damage and the need for RRT after severe failure of the kidneys, which raises few alarms in the initial stages. The majority of patients do not start RRT in a planned manner, so it is necessary to establish alternative methods for performing dialysis, primarily via central venous access on an urgent basis.^13^ In this context, creation of AVFs for dialysis patients is extremely important to continue treatment and they are also indicated because they reduce the risks associated with central venous catheters, such as bleeding, infections, and access failure, in addition to allowing patients greater autonomy.^15^

The current predominance of AVF access in the group studied herein underscores the importance of early planning of vascular access to maximize access survival, reduce adverse events, and improve patient quality of life.

Analysis of vascular access types showed that although the majority of patients started renal replacement therapy using short-duration catheters, a significant proportion migrated to native AVFs, which is in line with the national and international guidelines that recommend an AVF as the access of choice because of their better patency and lower rate of infectious complications.^6^

The elevated rate of initial catheter use may reflect difficulties with creation of AVFs in patients being cared for in ambulatory settings and still on conservative treatment. However, in accordance with the recommendations of current guidelines,^6,7^ progressive improvements in management of vascular accesses are being implemented, with a focus on proactive planning and on expanding access to specialist assessments, which has contributed to the increases in the proportion of definitive AVFs as follow-up progresses.

With relation to the complications associated with CVCs, the site of insertion influences the risk of infections and other complications related to local anatomy. Although there were no statistically significant differences in the current sample, it was observed that the preferred puncture site for access was on the right, because of the lower risk of injury to lymphatic structures and fewer changes to the anatomic course. In contrast, femoral artery punctures were rarely used, because of the increased risk of infection.^16,17^

Long-dwelling central lines and femoral puncture sites were associated with a greater risk of catheter infection and sepsis in intensive care unit settings.^18^

Table 8 shows the high prevalence of hospital admissions (67.5%) among patients on RRT in Pará, particularly hypertensive emergencies (77.1%) and infections associated with vascular access (33.3%), which were significant causes of hospital admissions. These data are in line with findings described by Harduin et al.,^6^ who stressed the need for rigorous surveillance of hemodialysis vascular accesses, aiming to minimize infectious complications and other adverse events that can compromise access patency, increase morbidity rates and, consequently, increase hospital admissions.^6,16-18^

The causal factor that led patients to RRT is a determinant factor in indicating kidney transplantation, together with other factors linked to prognosis and patients’ estimated life expectancy. Effective communication between health professionals and patients in conjunction with better institutional policies on live donors and opportune access to transplantation resources are essential factors that must be applied to the contingent identified in the study.^19,20^

Considering the findings related to the number and causes of admissions and complications during treatment, insufficient dialysis sessions, or fewer sessions than the number prescribed, can cause hypervolemic states, in which the primary manifestation is increased pressure, which is not necessarily accompanied by other congestive symptoms.^21,22^ Infections, which may or may not be related to access, were also a significant cause of admissions related to the path created for access or to an AVF, in combination with chronic disease immunocompromise.^22,23^

This study offers vascular specialists an unprecedented regional analysis, highlighting the elevated prevalence of initial hemodialysis via short duration catheters and later transition to stable and safe accesses, as recommended by current guidelines.^6^ The study also highlights the impact of comorbidities, such as hypertension and diabetes, on complications and admissions, emphasizing the need for strategies for early planning of vascular access and management of risk factors tailored to the epidemiological and health care specificities of the state of Pará.

This study has important limitations. First, as a single-center study conducted in the state of Pará, the generalizability of the findings to other epidemiological and health care settings should be interpreted with caution. Second, the sample size may not have been large enough to capture the entire diversity of cases and factors involved. Third, the retrospective design, based on extraction of data from medical records, also involves a risk of bias caused by possible incomplete or imprecise information. Finally, with regard to data specific to vascular access, significant differences between different types of access were not analyzed (whether by site or technique employed), which could have further enriched the discussion. As such, it is understood that there is a need for additional, wider- and deeper-ranging studies to provide more solid foundations that could guide adoption of effective clinical measures and therapeutic strategies.

## CONCLUSIONS

Patients on renal replacement therapy in Pará predominantly initiate hemodialysis in an unplanned manner, using temporary venous catheters, although they transition to definitive vascular access over time. This scenario highlights the need for early diagnosis of chronic kidney disease and timely vascular access planning, aiming to reduce complications and optimize clinical outcomes.

## DISPONIBILIDADE DE DADOS

Todos os dados gerados ou analisados estão incluídos neste artigo e/ou no material suplementar.
