# Supplementary material for: Vascular access in Pará: clinical and epidemiological aspects of patients on renal replacement therapy at the largest specialist center in Pará state, Brazil
Source: J Vasc Bras. 2026 Apr 27;25:e20240052. doi: 10.1590/1677-5449.202400522 (PMC13142166; doi:10.1590/1677-5449.202400522)
Supplement: Translation

# Translation: Acessos vasculares no Pará: aspectos clínico-epidemiológicos dos pacientes em terapia renal substitutiva no maior centro de referência do estado

**DOI:** 10.1590/1677-5449.202400521

**Authors:** José Maciel Caldas dos Reis, Iara de Brito Silva, Maria Gabriela Perdigão Barros Monteiro, Maria Mirella Nunes Bessa Guerra, Daniele Lima da Costa, Fabiany de Fátima Pompeu Rodrigues, Mariseth Carvalho de Andrade

**Keywords:** insuficiência renal, diálise renal, epidemiologia, terapia renal substitutiva

## Abstract

**Contexto:**

No estado do Pará, não há dados oficiais quanto à prevalência de doença renal crônica terminal em diálise.

**Objetivos:**

Descrever o perfil clínico-epidemiológico dos pacientes em hemodiálise no maior centro de referência do Pará.

**Métodos:**

Trata-se de um estudo observacional, transversal, descritivo e quantitativo. Foi realizada estatística descritiva simples. Os dados foram coletados por meio de prontuários, de junho a agosto de 2022, no Centro de Hemodiálise do Hospital de Clínicas Gaspar Vianna (HCGV) e na Clínica de Hemodiálise Monteiro Leite (CHML), ambos em Belém (PA). Foram entrevistados 191 pacientes do programa crônico de hemodiálise.

**Resultados:**

Dos entrevistados, 28,8% eram pacientes do HCGV e 71,6%, do CHML. Desses, 57,1% eram homens e 42,9%, mulheres. A média de idade foi de 54,1 anos. Entre os resultados observados, 65,4% se autodeclararam pardos, 44,5% completaram o ensino fundamental, 41,9% eram solteiros, 71,4% eram hipertensos, 40,6% eram diabéticos e 77,5% tinham média de 4 anos em terapia renal substitutiva. Em relação ao tratamento, 86,9% iniciaram a terapia por cateter de curta duração, 8,4% utilizaram o de longa permanência e 4,7% apresentaram o acesso definitivo com uma fístula arteriovenosa (FAV) maturada. Atualmente, 58,1% dos pacientes apresentam FAV nativa, 34% têm cateter de longa permanência e 3,1% possuem cateter de curta duração e sem FAV.

**Conclusões:**

Pacientes em terapia renal substitutiva no Pará iniciam a hemodiálise predominantemente de forma não planejada, por meio de cateteres venosos temporários, embora evoluam para acessos definitivos ao longo do seguimento. Esse cenário evidencia a necessidade de diagnóstico precoce da DRC e planejamento antecipado do acesso vascular, visando reduzir complicações e otimizar os desfechos clínicos.

## INTRODUÇÃO

A doença renal crônica (DRC) é um problema de saúde pública mundial, e suas principais causas são a hipertensão arterial e o diabetes melito (DM) nas mais diversas populações^1^ . Em 2012, a definição clínica da DRC foi atualizada pela Sociedade Internacional de Nefrologia como sendo a medida da taxa de filtração glomerular (TFG) abaixo de 60 mL/min/1,73 m^2^, por pelo menos 3 meses, independentemente da causa. A doença renal em estágio final ou doença renal crônica terminal (DRCT), por sua vez, ocorre quando a TFG estimada é menor que 15 mL/min/1,73 m^2^, indicando falência renal com necessidade de terapia dialítica^2^ .

A fase terminal da insuficiência renal crônica (IRC) é caracterizada pela deterioração das funções bioquímicas e fisiológicas de todos os sistemas do organismo, ocorrendo quando os rins são incapazes de remover os produtos de degradação metabólica do corpo ou de realizar as funções reguladoras^1-3^ .

O rastreamento associado ao tratamento adequado das doenças renais pode postergar a evolução e a progressão da perda das propriedades renais^1,2^ . O envelhecimento da população e o aumento da expectativa de vida, decorrentes da transição demográfica nas últimas décadas no Brasil, contribuíram para mudanças no perfil de morbimortalidade e aumento da prevalência das doenças crônicas, entre elas, a DRC^3,4^ .

Os cateteres venosos centrais (CVC) são componentes permanentes do processo da hemodiálise, sobretudo para os pacientes que realizam ciclos dialíticos três vezes por semana. No entanto, práticas e manuseios inadequados dos CVC contribuem para a instalação de processos infecciosos, tanto no sítio quanto de forma sistêmica, podendo levar a sérias complicações, como a septicemia^4,5^ .

No Brasil, os dados atualizados pelo Censo Brasileiro de Diálise 2020 confirmam a tendência observada nos últimos anos em relação ao aumento do número de pacientes em diálise, perfazendo um total estimado de pacientes da ordem de 144.779. Foram estimados 44.264 novos pacientes em diálise em 2020, sendo que a prevalência estimada de pacientes em diálise na região Norte do Brasil é de 283 partes por milhão da população (pmp), com taxa de incidência total de 209 pmp, superior à da América Latina (159 pmp) e da Europa (122 pmp) em 2018 e inferior à dos Estados Unidos (370 pmp) em 2017^1,5,6^ .

No estado do Pará, não há dados oficiais quanto à prevalência de DRCT com necessidade de diálise crônica. O levantamento do Plano Estadual de Atenção ao Portador de DRC, publicado em maio de 2015, apontou 2.692 pacientes em terapia renal substitutiva (TRS) no Pará, em 23 serviços de TRS cadastrados no Cadastro Nacional de Estabelecimentos de Saúde (CNES) naquele ano. Dessa forma, o atendimento foi prestado apenas para cerca de metade da população de pacientes que necessitavam de TRS, a despeito da ampliação da oferta de serviços de TRS e vagas de hemodiálise no mesmo estado a partir de 2011^4^ .

O crescente número de pacientes com DRC representa um importante desafio para o sistema de saúde, uma vez que, além de gerar custos progressivos à saúde pública, está associado a elevados índices de morbidade e mortalidade^7-9^ .

Assim, o objetivo do presente estudo é descrever o perfil clínico- epidemiológico dos pacientes em TRS no maior centro de referência em hemodiálise do estado do Pará.

## MÉTODOS

### Desenho do estudo

Trata-se de um estudo observacional, transversal, retrospectivo e descritivo, de abordagem quantitativa, e, em parte, de base documental e de campo. A metodologia aplicada seguiu os preceitos contidos no documento Guidelines On Good Publication Practice, elaborado pelo Committee on Publication Ethics (COPE)^10^ . Os dados foram coletados de junho a agosto de 2022, durante as sessões de hemodiálise, no Centro de Hemodiálise do Hospital de Clínicas Gaspar Vianna (HCGV) e na Clínica de Hemodiálise Monteiro Leite (CHML), ambos localizados em Belém (PA). As informações clínicas específicas foram captadas por meio dos prontuários disponíveis no local. Foram entrevistados 191 pacientes de um total de 214 pacientes no programa crônico de hemodiálise da instituição.

Os pacientes responderam a um questionário padrão semiestruturado com o auxílio de um pesquisador que coletou os dados nas unidades de hemodiálise. Todos os pacientes ou responsáveis legais preencheram o Termo de Consentimento Livre e Esclarecido (TCLE), permitindo a divulgação científica das informações.

### Aspectos éticos

O projeto foi submetido previamente ao Comitê de Ética em Pesquisa (CEP), com número do Certificado de Apresentação de Apreciação Ética (CAAE) 00497817.4.0000.5701, e seguiu a Resolução 466/2012 do Conselho Nacional de Saúde, preservando o sigilo e o anonimato. Foi aceito pelo CEP sob parecer substanciado n° 2.954.5541.

### População do estudo

Foram incluídos na pesquisa todos os pacientes renais crônicos dialíticos matriculados na instituição, que aceitaram participar do estudo e que assinaram o TCLE, maiores de 18 anos, de ambos os sexos, e que faziam parte do programa crônico de hemodiálise do HCGV e da CHML. Foram excluídos pacientes em diálise peritoneal e aqueles que, por algum motivo, não compareceram às sessões de hemodiálise nos dias das coletas de dados, conforme fluxograma baseado na diretriz STROBE demonstrado na Figura 1.

**Figura 1:** Fluxograma com seleção, perdas e inclusão de pacientes no estudo.

### Cálculo amostral

Para descrever o perfil clínico-epidemiológico dos pacientes em TRS no maior centro de referência do Pará, foi realizado cálculo amostral, considerando um nível de confiança de 95% (Z = 1,96), erro tolerável de 5% e proporção esperada de 50%. Com base na população atendida, 214 pacientes em TRS, o tamanho amostral final foi de 130 indivíduos, calculado conforme a metodologia proposta por Luchesa e Chaves^11^ .

### Dados coletados

A coleta de dados ocorreu com auxílio de um instrumento com questões estruturadas, contendo dados sociodemográficos (idade, sexo, estado civil, etnia e escolaridade) e clínicos (variáveis referentes às doenças de base relacionadas à hemodiálise e tipos de acessos) dos pacientes. A investigação das intercorrências hemodinâmicas, infecciosas e metabólicas relacionadas ao acesso vascular ou de outras complicações sistêmicas durante a hemodiálise considerou os eventos ocorridos nos 6 meses anteriores à data da coleta de dados.

### Análise estatística

Os dados foram analisados de forma criteriosa, com base em frequências absolutas e relativas (percentuais) para as variáveis categóricas. Para comparação entre proporções, foi utilizado o teste do qui-quadrado de Pearson. Foi adotado nível de significância de α = 0,05 (5%), sendo considerados estatisticamente significativos os valores de p < 0,05, os quais foram sinalizados com asterisco (*). A organização e análise dos dados foram realizadas com auxílio do programa Microsoft Excel®.

## RESULTADOS

A análise do perfil socioeconômico dos pacientes permite identificar os aspectos de sua realidade clínica e social. Foram investigados 191 pacientes, 55 (28,8%) do HCGV e 136 (71,6%) da CHML. Quanto ao sexo, 57,1% pacientes eram do sexo masculino e 42,9%, do feminino. No que diz respeito à faixa etária, a idade média foi de 54,1 anos, variando entre 18 e 93 anos. As duas maiores prevalências foram nas faixas etárias de 50 a 59 anos e 60 a 69 anos. A cor de pele autorreferida predominante foi parda (65,4%) (Tabela 1).

**Tabela 1:** Aspectos demográficos dos pacientes.

**Variáveis**	**N**	**%**
Sexo		
Feminino	82	42,9
Masculino	109	57,1
Faixa etária^*^ (anos)		
< 30	20	10,5
30 a 39	14	7,3
40 a 49	33	17,3
50 a 59*	47	24,6
60 a 69*	42	22,0
≥ 70	35	18,3
Etnia*		
Parda*	125	65,4
Negra	35	18,3
Branca	31	16,2

* p = 0,0001; teste qui-quadrado.

Quanto à escolaridade, 44,5% dos pacientes afirmaram possuir o ensino fundamental completo. Quanto à situação conjugal, solteiros corresponderam a 41,9% da amostra. Em relação à classe nutricional, 46,1% foram considerados na classe de peso adequado, com apenas 8,4% na classe de baixo peso. Dados do perfil econômico dos pacientes apontaram que 83,3% possuíam renda salarial mensal entre 1 e 3 salários-mínimos, e, entre os entrevistados, 48,4% recebiam auxílio-doença do governo. A grande maioria dos pacientes (94,3%) vivia com a família, 80,2% moravam em casa própria e 58,9% advinham da capital do estado do Pará. Notou-se relevância em relação aos pacientes que têm residência no interior do estado e região metropolitana, totalizando 41,1% (Tabelas 2 e 3).

**Tabela 2:** Aspectos relacionados a escolaridade, situação conjugal e classificação nutricional dos pacientes.

**Variáveis**	**N**	**%**
Escolaridade^*^		
Sem escolaridade	11	5,8
Fundamental incompleto	19	9,9
Fundamental completo*	85	44,5
Ensino médio	57	29,8
Ensino superior	19	9,9
Situação conjugal*		
Solteiro(a)*	80	41,9
Casado(a)*	76	39,8
Divorciado(a)	19	9,9
Viúvo(a)	16	8,4
Classificação nutricional*		
Baixo peso	16	8,4
Peso adequado*	88	46,1
Sobrepeso	56	29,3
Obesidade	22	11,5
Sem informação	9	4,7

* p = 0,0001; teste qui-quadrado.

**Tabela 3:** Caracterização de dados socioeconômicos dos pacientes.

**Variáveis**	**N**	**%**
Renda (SM)^*^		
Sem renda	4	2,1
Menor que 1	6	3,1
1 a 3*	160	83,3
Acima de 3	6	3,1
Não soube informar	15	7,8
Situação da renda*		
Recebe auxílio-doença*	93	48,4
Aposentado	81	42,2
Espera benefício previdenciário	33	17,2
Continua trabalhando	22	11,5
Situação de moradia*		
Mora com a família*	181	94,3
Mora sozinho	11	5,7
Situação da casa*		
Própria*	154	80,2
Alugada	27	14,1
Cedida	11	5,7
Procedência*		
Capital*	113	58,9
Região metropolitana	21	10,9
Interior	58	30,2
Deslocamento para hemodiálise*		
Oferecido pela prefeitura	69	36,1
Outros deslocamentos*	122	63,9

* p = 0,0001; teste qui-quadrado.

SM = salários-mínimos.

Entre as causas da DRC, 39,1% dos pacientes desenvolveram a doença por consequência de hipertensão arterial sistêmica (HAS) e 34,4%, por DM tipo 2. As doenças associadas mais prevalentes foram HAS e DM, correspondendo a 71,4% e 40,6%, respectivamente. Quanto aos fatores de risco, os principais foram o etilismo, o tabagismo e a obesidade. Entre os entrevistados, 85 (44,3%) faziam uso de bebida alcoólica e 67 (34,9%) já fizeram uso do tabaco (Tabela 4).

**Tabela 4:** Fatores de risco associados à doença renal crônica dos pacientes.

**Variáveis**	**N**	**%**
Causas da DRC^*^		
Hipertensão*	75	39,1
DM tipo 2*	66	34,4
Causa indeterminada	32	16,7
LES	10	5,2
Doença policística	8	4,2
Cálculo renal	8	4,2
Glomerulonefrite	4	2,1
Outras causas	49	25,5
Doenças associadas*		
HAS*	137	71,4
Diabetes melito	78	40,6
Doenças reumáticas	45	23,4
AVC	41	21,4
Cálculos renais	38	19,8
Dislipidemia	35	18,2
IAM	30	15,6
Glomerulonefrite	18	9,4
Uropatias obstrutivas	12	6,3
Câncer	2	1,0
Fatores de risco		
Etilismo	85	44,3
Tabagismo	67	34,9
Obesidade	26	13,5

* p = 0,0001; teste qui-quadrado.

AVC = acidente vascular cerebral; DM = diabetes melito; DRC = doença renal crônica; HAS = hipertensão arterial sistêmica; IAM = infarto agudo do miocárdio; LES = lúpus eritematoso sistêmico.

É importante salientar que 169 (88,5%) pacientes não realizaram biópsia, e apenas 49 (25,7%) realizaram tratamento conservador, sendo que apenas 26,5% o fizeram por menos de 6 meses, 30,6% de 6 a 12 meses e 40,9% por mais de 1 ano.

Identificou-se que a maioria dos pacientes iniciava a TSR por meio de cateteres duplo-lúmen (CDL) de curta duração. Além disso, foi observado que a maioria dos pacientes evoluiu para fístula arteriovenosa (FAV) ou CVC de longa duração, a depender da indicação de TSR e das condições clínicas dos pacientes. As FAVs são a forma preferencial para os pacientes que necessitam de TSR por tempo indeterminado. Não foram avaliadas diferenças entre o local das FAVs ou número de procedimentos realizados (Tabelas 5 e 6).

**Tabela 5:** Modalidades de acesso venoso no início e final do estudo nos pacientes em terapia renal substitutiva no Pará.

**Variáveis**	**N**	**%**
Curta duração-CDL^*^	166	86,9
Longa duração-Permicath	16	8,4
FAV	9	4,7
Acesso vascular atual*		
FAV nativa*	111	58,1
Longa duração	65	34,0
FAV com PTFE	9	4,7
Curta duração-CDL	6	3,1

* p = 0,0001; teste qui-quadrado.

CDL = cateter duplo-lúmen; FAV = fístula arteriovenosa; PTFE = politetrafluoroetileno.

**Tabela 6:** Local e modalidade de acessos nos pacientes em terapia renal substitutiva no Pará.

**Variáveis**	**N**	**%**
Local CDL		
VSC Direita	3	50,0
VJI Direita	2	33,3
VFC Direita	2	33,3
Número de CDL realizados		
Um	1	16,7
Dois	4	66,7
Três ou mais	1	16,7
Local cateter longa duração^*^ (n=65)		
VJI Direita*	23	35,4
VSC Direita	17	26,2
VSC Esquerda	11	16,9
VJI Esquerda	8	12,3
VFC Esquerda	5	7,7
VFC Direita	1	1,5
Número de cateteres de longa duração realizados		
Um*	36	55,4
Dois	15	23,1
Três	6	9,2
Quatro ou mais	8	12,3
Tipo FAV* (n=120)		
BC Esquerdo*	44	36,7
BC Direito*	34	28,3
BB Esquerdo	18	15,0
RC Esquerdo	17	14,2
BB Direito	10	8,3
RC Direito	6	5,0
Número de FAVs realizadas*		
Um*	82	67,2
Dois	25	20,5
Três	6	4,9
Quatro ou mais	6	4,9

* p = 0,0001; teste qui-quadrado.

BB = braquiobasílica; BC = braquiocefálica; CDL = cateter duplo-lúmen; FAV = fístula arteriovenosa; RC = radio-cefálica; VFC = veia femoral comum; VJI = veia jugular interna; VSC = veia subclávia.

A maioria dos pacientes avaliados na amostra possuía interesse em transplante renal, apesar de não ser indicado a todos os casos (Tabela 7). Em relação às internações, aproximadamente 68% dos pacientes tiveram necessidade de internação, sendo urgências hipertensivas as principais causas relatadas (Tabela 8).

**Tabela 7:** Avaliação de fatores relacionados ao transplante renal e entendimento da doença.

**Variáveis**	**N**	**%**
Interesse em transplante renal^*^	125	65,4
Em preparação para transplante	69	36,1
Conhecimento prévio da doença	42	22,0
Antecedentes familiares de diálise	37	19,4

* p = 0,0001; teste qui-quadrado.

**Tabela 8:** Internações e causas entre pacientes em terapia renal substitutiva no Pará.

**Variáveis**	**N**	**%**
Internações hospitalares^*^		
Sim*	129	67,5
Não	42	22,0
Causa da internação* (n=129)		
Urgências hipertensivas*	108	77,1
Outras infecções	86	44,8
Distúrbios metabólicos	77	40,1
Infecção relacionada ao acesso	64	33,3
Eventos cardiovasculares	50	26,0
Hipervolemia	25	13,0

* p = 0,0001; teste qui-quadrado.

## DISCUSSÃO

O presente estudo analisou o questionário instituído pelos pesquisadores, respondido por 191 pacientes voluntários incluídos no programa crônico de hemodiálise do HCGV e da CHML. A análise dos dados demonstrou maior prevalência do sexo masculino, achado que está em consonância com a literatura, a qual associa esse predomínio a maior exposição a fatores de risco modificáveis, como etilismo, tabagismo, obesidade e sedentarismo, além de menor adesão às medidas preventivas e de promoção da saúde quando comparado ao sexo feminino^9,12^ .

Em relação à idade média encontrada entre os pacientes, o achado está em consonância com o perfil etário descrito no Censo da Sociedade Brasileira de Nefrologia de 2020, que demonstra maior concentração de indivíduos em faixas etárias mais avançadas entre os pacientes em TRS^1^ . No que diz respeito à cor da pele, os resultados devem ser interpretados à luz das características demográficas regionais, especialmente considerando a composição populacional predominante no estado do Pará e na região Norte^4^ .

A escolaridade prevalente demonstra que a condição de acesso educacional em relação ao nível de conhecimento da doença favorece a busca por tratamento precoce. Entende-se, também, que os pacientes com diagnóstico de DRC necessitam de tratamento muitas vezes prolongado, o qual tende a se tornar desgastante para o paciente e para sua rede de apoio, justificando o maior quantitativo de solteiros em tratamento^12^ .

A manutenção do peso adequado durante a terapia, dado identificado na maioria dos pacientes, é um fator positivo para a redução de complicações e internações hospitalares, sobretudo quando se sabe que a TRS implica de forma deletéria no metabolismo, principalmente nas proteínas, podendo acarretar desnutrição e piora do desfecho clínico^13^ .

A renda declarada corresponde à média mensal observada na população brasileira em 2022, conforme dados da Pesquisa Nacional por Amostra de Domicílios (PNAD) Contínua do Instituto Brasileiro de Geografia e Estatísticas (IBGE)^14^ , sobretudo com o incentivo financeiro do governo, cujo valor contribui para complementar a renda e auxiliar em custos básicos. Viver com a família auxilia no suporte emocional e logístico para manter o longo tratamento dialítico em atividade.

Dados do Censo da Sociedade Brasileira de Nefrologia demonstram que a HAS e o DM permanecem como as principais causas de DRC no Brasil^1^ . Esse perfil etiológico é consistente com a literatura nacional e internacional, que reconhece essas condições como os principais determinantes da progressão da DRC e da necessidade de TRS^3,9^ . Além disso, a elevada prevalência de doenças cardiovasculares e metabólicas na população contribui para a manutenção desse padrão epidemiológico.

Entre os fatores de risco, a ingestão de bebida alcoólica, realizada por cerca de metade dos entrevistados, quando consumida em doses elevadas, pode causar maior ingestão de energia e, assim como o excesso de peso, aumentar a pressão arterial e o risco cardiovascular, o que corrobora a progressão da DRC. Da mesma forma, o consumo do tabaco está associado ao aumento da albuminúria, que agrava a lesão renal progressivamente^6^ .

O início do tratamento com CDL de curta duração é notável devido à evolução oligossintomática da injúria renal e da necessidade de TSR após falência grave do órgão, pouco alarmada nas fases iniciais. A maioria dos pacientes não inicia a TSR de forma planejada, sendo necessário estabelecer outras formas de diálise, principalmente por meio de acessos venosos centrais em caráter de urgência^13^ . Dessa forma, a instituição da FAV nos pacientes dialíticos é de suma importância para a continuidade do tratamento, sendo indicada também para a diminuição dos riscos associados aos cateteres venosos centrais, como sangramento, infecções e falha do acesso, além de garantir mais autonomia ao paciente^15^ .

A predominância atual de FAVs no grupo estudado reforça a importância do planejamento precoce do acesso vascular para maximizar a sobrevida do acesso, reduzir eventos adversos e melhorar a qualidade de vida dos pacientes.

A análise dos tipos de acesso vascular evidenciou que, apesar da maioria dos pacientes iniciar a TRS por meio de cateteres de curta duração, houve uma progressão significativa para FAVs nativas, o que está em consonância com as diretrizes nacionais e internacionais que recomendam a FAV como acesso de escolha devido à sua maior perviedade e menor taxa de complicações infecciosas^6^ .

O uso inicial elevado de cateteres pode refletir as dificuldades na realização de FAV em pacientes acompanhados ambulatorialmente ainda em tratamento conservador. No entanto, de acordo com as atuais recomendações de diretrizes^6,7^ , melhorias progressivas no gerenciamento dos acessos vasculares vêm sendo implementadas, com foco no planejamento antecipado e na ampliação do acesso a avaliações especializadas, o que contribuiu para o aumento na proporção de FAVs definitivas ao longo do seguimento.

Entre as complicações associadas aos CVCs, seu local de inserção influencia no risco de infecções e outras complicações relacionadas à anatomia local. Apesar da não identificação de diferenças estatisticamente significativas na amostra em questão, observou-se que o local preferencial de punção foram os acessos à direita, em decorrência do menor risco de lesões em estruturas linfáticas e menos alterações de percurso anatômico. Por outro lado, as punções da artéria femoral foram pouco observadas por conta do maior risco infeccioso da região^16,17^ .

A longa permanência de cateteres centrais e o sítio de punção femoral foram associados ao maior risco de infecção do cateter e sepse em ambiente de unidade de terapia intensiva^18^ .

Nossos achados evidenciam alta prevalência de internações hospitalares (67,5%) em pacientes submetidos a TRS no Pará, destacando-se urgências hipertensivas (77,1%) e infecções relacionadas ao acesso vascular (33,3%) como importantes causas de hospitalização. Esses dados estão alinhados aos descritos por Harduin et al.^6^ , que enfatizam a necessidade de uma vigilância rigorosa dos acessos vasculares para hemodiálise, visando minimizar complicações infecciosas e outros eventos adversos capazes de comprometer a perviedade dos acessos, aumentar as taxas de morbidade e, consequentemente, elevar os índices de internação hospitalar^6,16-18^ .

O fator causal que leva os pacientes à TSR é determinante para indicação de transplante renal, assim como outros fatores relacionados ao prognóstico e à estimativa de vida do paciente. Comunicação efetiva entre os profissionais de saúde e os pacientes, assim como maiores políticas das instituições acerca de doadores vivos e acesso oportuno aos meios de transplante são aspectos necessários a serem aplicados ao contingente identificado na pesquisa^19,20^ .

Diante dos dados encontrados em relação ao número e ao motivo das internações e intercorrências durante o tratamento, diálises insuficientes ou em número abaixo do prescrito podem gerar um estado de hipervolemia, em que a principal manifestação é o aumento pressórico, sem necessariamente acompanhar outros sintomas congestivos^21,22^ . As infecções, relacionadas ou não ao acesso, também configuram importantes causas de internações em decorrência do pertuito gerado pelo acesso ou à FAV associada ao imunocomprometimento de doenças crônicas^22,23^ .

Este estudo oferece ao especialista vascular uma análise regional inédita, destacando a elevada prevalência do início da hemodiálise por cateteres de curta duração e a posterior transição para acessos estáveis e seguros aos pacientes, conforme recomendado pelas atuais diretrizes^6^ . A pesquisa também reforça o impacto de comorbidades, como hipertensão e diabetes, nas complicações e internações, ressaltando a necessidade de estratégias de planejamento precoce do acesso vascular e manejo de fatores de risco adaptados às especificidades epidemiológicas e assistenciais do estado do Pará.

O estudo apresenta importantes limitações. Em primeiro lugar, por tratar-se de um estudo unicêntrico realizado no estado do Pará, a generalização dos resultados para outros contextos epidemiológicos e assistenciais deve ser interpretada com cautela. Em segundo lugar, o tamanho da amostra pode não ser suficiente para representar a totalidade da diversidade de casos e fatores envolvidos. Em terceiro lugar, o desenho retrospectivo, baseado na coleta de dados de prontuários, também implica risco de viés devido a possíveis informações incompletas ou imprecisas. Por fim, no que se refere aos dados específicos de acesso vascular, não foram analisadas diferenças significativas entre os tipos de acesso (localização ou técnicas empregadas), o que poderia enriquecer ainda mais a discussão. Assim, entende-se que há uma necessidade de estudos futuros mais amplos e aprofundados para oferecer bases mais sólidas que possam orientar a adoção de medidas clínicas e de estratégias terapêuticas eficazes.

## CONCLUSÃO

Pacientes em terapia renal substitutiva no Pará iniciam a hemodiálise predominantemente de forma não planejada, por meio de cateteres venosos temporários, embora evoluam para acessos definitivos ao longo do seguimento. Esse cenário evidencia a necessidade de diagnóstico precoce da DRC e planejamento antecipado do acesso vascular, visando reduzir complicações e otimizar os desfechos clínicos.

## Data Availability

All data generated or analyzed are included in this article and/or in the supplemental material.
